# Distribution and clinical significance of anti-carbamylation protein antibodies in rheumatological diseases among the Chinese Han population

**DOI:** 10.3389/fimmu.2023.1197458

**Published:** 2023-07-19

**Authors:** Rongrong Dong, Yuanyuan Sun, Wei Xu, Weizhen Xiang, Meiqi Li, Qingrui Yang, Ling Zhu, Zhenzhen Ma

**Affiliations:** ^1^ Department of Rheumatology and Immunology, Shandong Provincial Hospital, Cheeloo College of Medicine, Shandong University, Jinan, Shandong, China; ^2^ Department of Respiratory and Critical Care Medicine, Shandong Provincial Hospital Affiliated to Shandong First Medical University, Jinan, Shandong, China; ^3^ Department of Rheumatology and Immunology, Shandong Provincial Hospital Affiliated to Shandong First Medical University, Jinan, Shandong, China

**Keywords:** rheumatic diseases, autoantibodies, anti-carbamylation protein antibodies, interstitial lung disease, Chinese patients

## Abstract

**Objective:**

Several studies have demonstrated that anti-carbamylation protein antibodies (Anti-CarPA) are persistent in patients with rheumatoid arthritis (RA), systemic lupus erythematosus (SLE), systemic sclerosis (SSC), primary Sjögren’s syndrome (pSS), and interstitial lung disease associated with RA (RA-ILD). However, the relationship between anti-CarPA and other rheumatic diseases (RDs) and non-RA-ILD is not known till now. This study sought to examine the presence of anti-CarPA in Chinese Han patients with RDs and its clinical significance.

**Methods:**

The study included 90 healthy controls (HCs) and 300 patients with RDs, including RA, SLE, polymyositis/dermatomyositis (PM/DM), pSS, SSC, spondyloarthritis (SpA), anti-neutrophil cytoplasmic autoantibodies associated with vasculitis (AAV), undifferentiated connective tissue disease (UCTD), and Behcet’s disease (BD). Antibodies against carbamylated human serum albumin were detected using commercial enzyme-linked immunosorbent assay kits. Correlations between clinical and laboratory parameters were analyzed.

**Result:**

Serum levels of anti-CarPA in RA (34.43 ± 33.34 ng/ml), SLE (21.12 ± 22.23 ng/ml), pSS (16.32 ± 13.54 ng/ml), PM/DM (30.85 ± 17.34 ng/ml), SSC (23.53 ± 10.70 ng/ml), and UCTD (28.35 ± 21.91 ng/ml) were higher than those of anti-CarPA in the HCs (7.30 ± 5.05 ng/ml). The concentration of serum anti-CarPA was higher in patients with rheumatic disease-related interstitial lung disease (RD-ILD), especially RA-ILD, PM/DM-ILD, and pSS-ILD. Patients with RD-ILD who tested positive for anti-CarPA were more likely to have a more severe radiographic classification (grades II, p = 0.045; grades III, p = 0.003). Binary logistic regression analysis suggested that anti-CarPA had an association with ILD in RA (p = 0.033), PM/DM (p = 0.039), and pSS (p = 0.048). Based on receiver operating characteristics (ROC) analysis, anti-CarPA cutoffs best discriminated ILD in RA (>32.59 ng/ml, p = 0.050), PM/DM (>23.46 ng/ml, p = 0.038), and pSS (>37.08 ng/ml, p = 0.040). Moreover, serum levels of anti-CarPA were correlated with antibodies against transcription intermediary factor 1 complex (anti-TIF1) (R = –0.28, p = 0.044), antibodies against glycyl-transfer ribonucleic acid synthetase (anti-EJ) (R = 0.30, p = 0.031), and antibodies against melanoma differentiation-associated gene 5 (anti-MDA5) (R = 0.35, p = 0.011).

**Conclusion:**

Serum anti-CarPA could be detected in patients with RA, PM/DM, pSS, SSC, and UCTD among the Chinese Han population. And it may also assist in identifying ILD in patients with RA, PM/DM, and pSS, which emphasized attention to the lung involvement in anti-CarPA-positive patients.

## Introduction

Rheumatic diseases (RDs) are characterized by long disease duration, diverse clinical manifestations, and various prognoses. The diagnosis of these diseases is primarily determined by the clinical manifestations and the presence of specific autoantibodies. In clinical practice, early diagnosis of RDs among some patients is challenging due to the absence of specific autoantibodies. Exploration of new antibodies is essential in the study of RDs. Anti-carbamylated protein antibody (anti-CarPA) is one of the new autoantibodies discovered in recent years. According to some studies, patients with RDs always have higher levels of anti-CarPA than healthy individuals. Indeed, antibodies to anti-CarPA are widely used in rheumatological research in RA and have been demonstrated to be associated with its diagnostic efficiency (1), risk stratification (2, 3), and treatment evaluation of RA patients (4), making it an ideal biomarker.

Carbamylation is a non-enzymatic process by which self-proteins are added to a cyanate group. In this process, lysine is converted to a homo-citrulline in the tertiary structure (5). Several proteins in the body, including albumin, low density lipoprotein, fibrinogen, enolase, 78 kDa glucose regulatory protein, vimentin, and α-1 antitrypsin, could be carbamylated (6–10). Many physiological and pathological processes, such as aging, cataracts, atherosclerosis, chronic kidney disease, and nervous system disorders, are also affected by carbamylated proteins (11). Additionally, some studies have suggested that the positive charge is inhibited after protein carbamylation, changing the interactions among ions on the protein surface. During these processes, the secondary and/or tertiary structure of proteins could be changed, exposing the abnormal region of the protein, thereby producing anti-CarPA (11, 12). Initially described in 2011 among patients with RDs, anti-CarPA could recognize homocitrullinated peptides (13). In subsequent studies, anti-CarPA was found in non-RA RDs, such as systemic lupus erythematosus (SLE), systemic sclerosis (SSC), and primary Sjögren’s syndrome (pSS), with different outcomes (14–20). Studies on anti-CarPA have revealed that it is associated with poor disease outcomes, including increased disease activity, radiographic progression, and mortality in patients with RA (1–3, 21, 22). Recent studies have also linked anti-CarPA with RA associated interstitial lung disease (RA-ILD), which may explain the increased mortality rate among anti-CarPA-positive patients with RA (2, 23).

Rheumatic disease-related interstitial lung disease (RD-ILD) is a common complication in patients with RDs. Depending on the screening method and the sample population, ILD affects 3–70% of patients with RDs. Difficult diagnosis, poor prognosis, and lack of effective treatments made RD-ILD one of the main causes of death in patients with RDs, thus prompting greater efforts to detect the disease as early as possible. However, clinically effective biomarkers for RD-ILD are still lacking, and early detection is problematic. A number of risk factors have been identified in patients with RD-ILD; they include smoking, male gender, higher disease activity, longer disease duration, older age, positive rheumatoid factor (RF), and anticitrullinated protein antibodies (ACPAs) (23, 24). Several biomarkers have also been proposed for RD-ILD screening, but none has been universally accepted. Recent studies have identified anti-CarPA as a potentially useful biomarker for RA-ILD, but other RD-ILDs have not been studied yet. Besides, the clinical significance in RDs other than RA was still unclear. Therefore, we sought to determine the presence of anti-CarPA in Chinese Han patients with RDs and its clinical significance. In this study, serum anti-CarPA levels and the association between anti-CarPA and its clinical significance including RD-ILD were also investigated among a diverse population of patients with RDs.

## Materials and methods

### Study population

**Figure 1:**
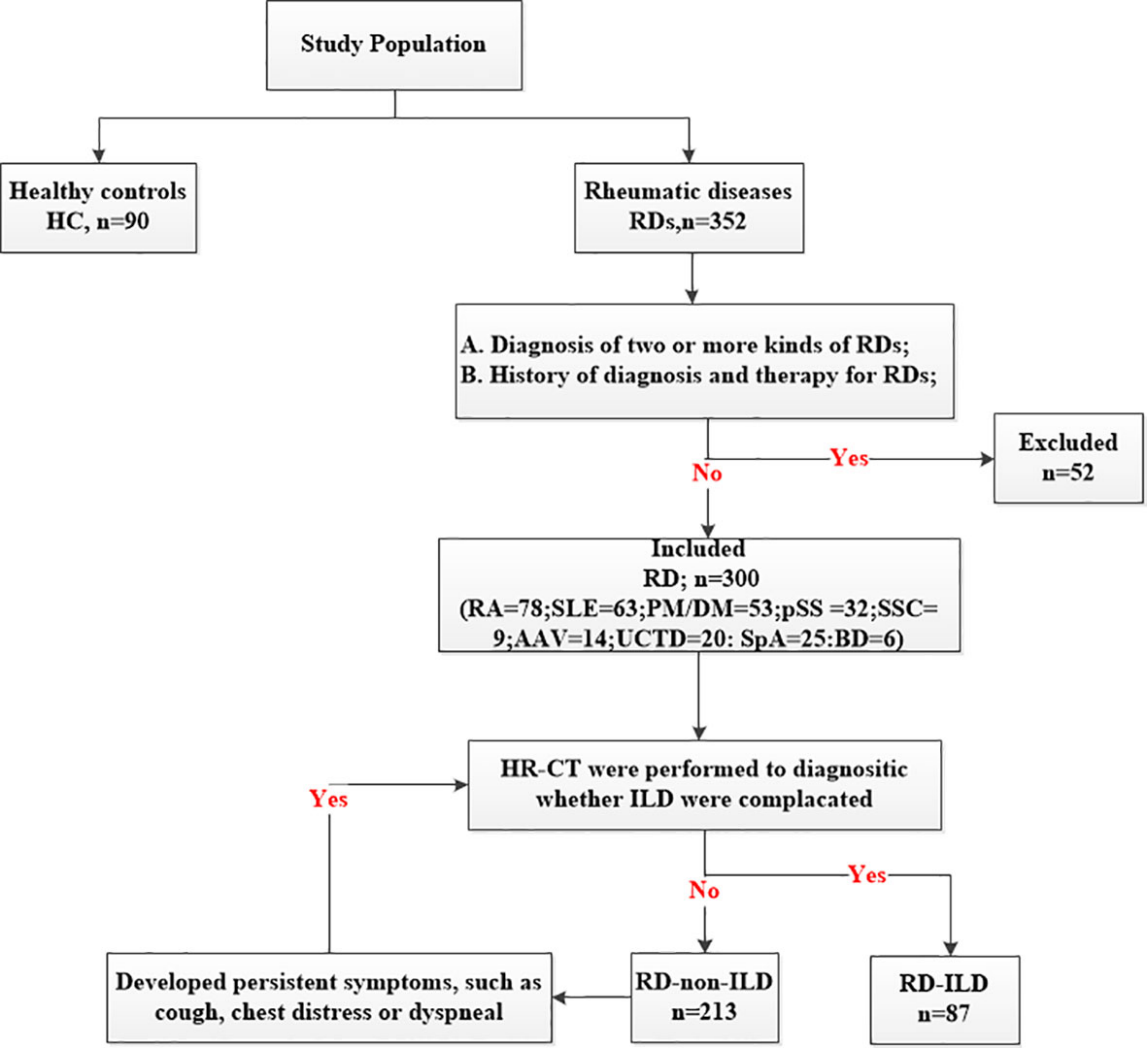
Study cohort.

This retrospective study involved 300 patients and 90 healthy controls (HC) consecutively admitted to the Rheumatology Department, Shandong Provincial Hospital from November 2020 to January 2022. All of patients were Han population. The inclusion criterion was diagnosis of one of the RDs, defined according to the updated international classification criteria, which is as followed: RA (25): American College of Rheumatology/European League Against Rheumatism (ACR/EULAR) classification criteria in 2010; SLE (26): The Systemic Lupus International Collaborating Clinics (SLICC) criteria for SLE in 2012; PM/DM (27): diagnostic criteria proposed by Bohan and Peter in 1975; pSS (28): ACR classification criteria in 2012; AAV (29): ACR classification criteria in 1990; SSC (30): ACR/EULAR classification criteria in 2013; SpA (31):International Spondyloarthritis Assessment Association SpA classification criteria in 2009; UCTD (32): definition proposed by Marta Mosca in 2014; BD (33): International Criteria for Behçet’s Disease in 2014. The exclusion criteria included (1) diagnosis of two or more kinds of RDs and (2) history of diagnosis and therapy for RDs (**Figure 1**). Furthermore, we divided the participants into subgroups based on whether they had ILD or not. The high-resolution computed tomography (HR-CT) was performed on all patients. If patients without ILD developed persistent symptoms, such as cough, chest distress or dyspneal, they would be suspected of ILD and repeated HR-CT. The patterns of ILD were classified by HR-CT, which was reviewed by two experienced radiologists according to the ATS/ERS International Multidisciplinary Consensus Classification of the Idiopathic Interstitial. According to the HR-CT manifestations of RD-ILD, patients were classified into three types: usually interstitial pneumonia (UIP), non-specific interstitial pneumonia (NSIP), and unclassifiable (34). The classification was based on clinical criteria for the definition of idiopathic interstitial pneumonia by the Japanese Ministry of Health and Welfare (35). The classifications included grades I (the lesion range does not exceed the peripheral lung field), II (the lesion range exceeds the peripheral lung field but does not exceed the medial lung field), and III (the lesion range exceeds the medial lung field). The local ethics committee of Shandong Provincial Hospital validated the study protocol, and all participants provided informed consent before enrolment (SZRJJ: NO.2021-438).

### Detection of antibodies against carbamylated human serum albumin

Autoantibody status was measured in sera collected at study enrolment. We used a commercial human anti-CarPA enzyme-linked immunosorbent assay (ELISA) kit (Fine Test, Wu Han, China) to measure anti-CarPA levels in patients with RDs and HCs. This kit quantifies all isotypes of anti-CarPA. After dialyzing at 4°C against distilled water for 36 hours, all reaction mixtures were stored at 20°C. The manufacturer’s instructions were followed, and the detailed procedure was as follows. ELISA microplates were coated with carbamylated human serum albumin and washed with the wash buffer. Sera were diluted with sample dilution buffer at a ratio of 1:2 and subsequently added to the ELISA wells (300 ul/well) for 90 minutes at 37°C. After washing with the wash buffer, biotinylated secondary antibodies were added to the ELISA wells (100 ul/well) for 60 minutes at 37°C. After additional washing with wash buffer, strept avidin-biotin complex (SABC) was added to the ELISA wells (100 ul/well) for 30 minutes at 37°C. The reaction was developed with tetramethylbenzidine (TMB) (90 ul/well) for 15 minutes at 37°C and then terminated with the stop solution (50 ul/well). Optical density (OD) was measured at 450 nm using an ELISA spectrophotometer (ThermoMultiskan GO Type:1510). Serum anti-CarPA levels were expressed in ng/ml. The detection limit of anti-CarPA was 0.781-50ng/ml, and the sensibility was 0.469ng/ml.

## Collection of clinical and laboratory indices

Beside the level of anti-CarPA, the following clinical information of RD patients was collected directly from the medical record (all of patients were hospitalized): rheumatoid factor (RF), anti-cyclic citrullinated peptide antibodies (CCP), antinuclear antibody (ANA), anti-Smith antibodies (SM),anti-double-stranded DNA antibody (dsDNA), antibodies reactive against the ribonucleoprotein antigens Ro/Sjögren’s syndrome A antigen (SSA), antibodies reactive against the ribonucleoprotein antigens La/Sjögren’s syndrome B antigen (SSB), and myositis-specific autoantibodies (MSAs). RF was detected by immune turbidimetry (0–20KU/L). SSA, SSB, and dsDNA were measured by ELISA with the normal range: SSA (0–20 RU/ml), SSB (0–20 RU/ml), and dsDNA (0–100 IU/ml). ANA was detected by indirect immunofluorescence. CCP and SM were measured by chemiluminescent immunoassay with the normal range: CCP (0–20 U/ml) and SM (0–20 CU). MSAs were detected using line blot techniques.

## Statistical analysis

Data analyses were performed using SPSS 26 software for Windows (IBM SPSS Inc., Chicago, USA) and GraphPad Prism 9 for Windows (San Diego, CA, USA). Results from parametric data were expressed as mean SD, and differences between groups were analyzed using a student’s T test. For nonparametric data, results were expressed as median (interquartile range) values, and differences between groups were analyzed using the Mann-Whitney test and Kruskal and Willis test. A correlation analysis between two continuous variables was performed using Spearman’s analysis. Multivariable analysis was then used to compare variables that had a p-value <0.1 by single-variable analysis. For all statistical analyses, p-values <0.05 were considered statistically significant.

## Results

### Baseline characteristics of the study population

A total of 300 patients with RDs were enrolled in the main study. They included 78 patients with RA, 63 patients with SLE, 53 patients with PM/DM, 32 patients with pSS, 14 patients with anti-neutrophil cytoplasmic autoantibodies (ANCA)-associated vasculitis (AAV), 9 patients with SSC, 20 patients with undifferentiated connective tissue disease (UCTD), 6 patients with Behcet’s disease (BD), and 25 patients with spondyloarthritis (SpA). The diseases were further grouped into ILDs and non-ILDs: RA (12 vs 66), SLE (14 vs 49), PM/DM (38 vs 15), pSS (7 vs 25), AAV (4 vs 10), SSC (5 vs 4), and UCTD (5 vs 15). There were no ILD patients in SpA group and BD group, so we didn’t set up ILD sub-group in them. Patients in this study were predominantly female (230/300) with a gender ratio of 76.67%, mean age of 50.46 ± 15.75 years, disease duration of 12.00 (3.00, 60.00) months, and smoking percentage of 12.00% (36 patients). Ninety HCs, comprising 25 men and 65 women, were included; they had a mean age of 46.32 ± 15.74 years. No significant differences were observed between the patients and HCs at baseline (**Table 1**).

**Table 1:** Demographic and clinical characteristics of the 300 patients with RDs and 90 Healthy controls.

Disease	Positive rate of anti-CarPA (%)	n	Gender F/M	Age(years) mean ± SD	Disease duration (month), median(IQR)/mean ± SD	Smoker (n(%))
RA	48.71	78	62/16	54.96 ± 14.47	9.50(3.00,60.00)	7/78(8.97%)
RA	+	38	29/9	54.71 ± 15.80	10.50(2.00,84.00)	4/38(10.53%)
RA	–	40	33/7	55.20 ± 13.29	9.50(4.00,48.00)	3/40(7.50%)
SLE	28.57	63	57/6	41.35 ± 16.07	12.00(5.00,60.00)	4/63(6.35%)
SLE	+	18	16/2	47.72 ± 15.20	30.00(2.75,126.00)	2/18(11.11%)
SLE	–	45	41/4	38.80 ± 15.85	12.00(6.00,36.00)	2/45(4.44%)
PM/DM	62.26	53	35/18	53.64 ± 12.73	6.00(2.00,12.00)	13/53(24.53%)
PM/DM	+	34	23/11	53.22 ± 10.54	8.00(2.00,24.00)	8/33(24.24%)
PM/DM	–	19	12/7	54.44 ± 16.50	3.50(2.00,12.00)	5/19(26.32%)
pSS	31.25	32	32/1	53.18 ± 13.84	30.00(6.00,84.00)	0
pSS	+	10	9/1	51.90 ± 12.92	12.00(6.00,93.00)	0
pSS	–	22	22/0	53.77 ± 14.50	36.00(5.00,84.00)	0
AAV	21.42	14	9/5	62.71 ± 9.90	6.00(4.75,9.50)	3/14(21.43%)
AAV	+	3	3/0	62.33 ± 11.50	5.67 ± 2.08	0
AAV	–	11	6/5	62.81 ± 10.04	6.00(5.00,12.00)	3/11(27.27%)
SSC	44.44	9	8/1	50.78 ± 12.59	73.89 ± 12.49	1/9(11.11%)
SSC	+	4	4/0	55.75 ± 15.88	75.00 ± 35.83	0
SSC	–	5	4/1	46.80 ± 9.15	73.00 ± 42.90	1/5(20.00%)
UCTD	55.00	20	14/6	50.10 ± 16.72	12.00(2.25,45.00)	4/20(20.00%)
UCTD	+	11	8/3	48.18 ± 18.07	6.00(1.70,24.00)	3/11(27.27%)
UCTD	–	9	6/3	52.44 ± 15.65	24.00(7.50,54.00)	1/9(11.11%)
SpA	24.00	25	9/16	44.08 ± 17.33	2.00(0.85,48.00)	4/25(16.00%)
SpA	+	6	4/2	43.00 ± 19.54	2.00(0.88,123.00)	1/6(16.67%)
SpA	–	19	5/14	44.42 ± 17.14	6.00(0.70,24.00)	3/19(15.79%)
BD	0	6	5/1	43.83 ± 7.44	60.00(0.88,174.00)	0
TOTAL	41.00	300	230/70	50.46 ± 15.75	12.00(3.00,57.00)	36/300(12.00%)
HC	–	90	65/25	46.32 ± 15.74	–	17/90(18.89%)

SD, standard deviation; IQR, interquartile range; RDs, Rheumatic diseases; RA, rheumatoid arthritis; SLE, systemic lupus erythematosus; PM/DM, polymyositis/dermatomyositis; pSS, primary Sjögren’s syndrome; AAV, anti-neutrophil cytoplasmic autoantibodies associated vasculitis; SSC, systemic sclerosis; UCTD, undifferentiated connective tissue disease; BD, Behcet’s disease; SpA, spondyloarthritis; HC, healthy controls.

Parametric data results were expressed as mean ± SD values; Nonparametric data results were expressed as median (IQR) values. Some values presented as n or n(%); n represents the number of people according with the criteria. Student’s -test was used in comparison between parametric data. Mann-Whitney test was used in comparison between parametric data and nonparametric data or nonparametric data.

### Clinical features of patients with RDs and the distribution of anti-CarPA

**Figure 2:**
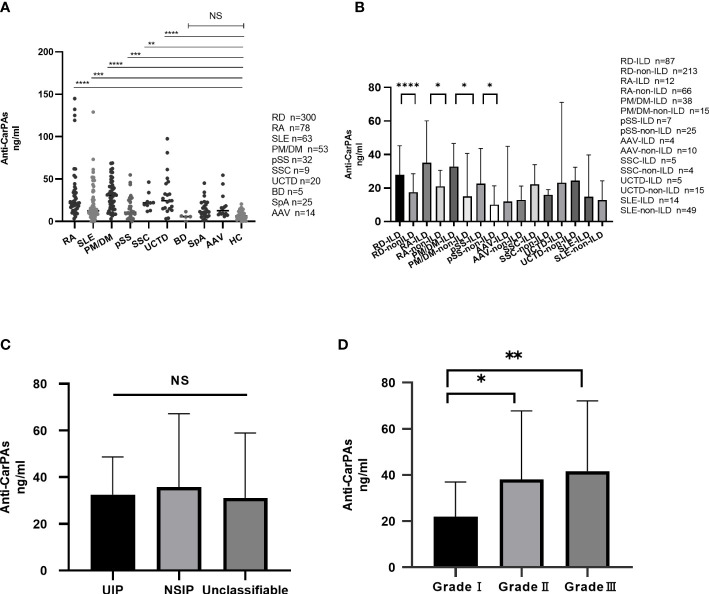
**(A)** Distribution of anti-CarPA in RDs. Serum sample obtained from 78 patients with rheumatoid arthritis (RA), 63 patients with systemic lupus erythematosus (SLE), 53 patients with polymyositis/dermatomyositis (PM/DM), 32 patients with primary Sjögren’s syndrome (pSS), 14 patients with anti-neutrophil cytoplasmic autoantibodies associated vasculitis (AAV), 9 patients with systemic sclerosis (SSC), 20 patients with undifferentiated connective tissue disease (UCTD), 6 patients with Behcet’s disease (BD), 25 patients with spondyloarthritis (SpA), and 90 healthy controls. Serum levels of anti-CarPA in RDs patients was measured by ELISA. Kruskal-Wallis rank sum test were used in multiple samples; Dunnett’s multiple comparisons test was used for pairwise comparisons. **(B)** Distribution of anti-CarPA in RD-ILD. Mann-Whitney test used for pairwise comparisons. **(C)** Distribution of anti-CarPA in different image classifications of RD-ILD. Kruskal-Wallis rank sum test were used in multiple samples; Bonferroni adjustment used for pairwise comparisons. **(D)** Distribution of anti-CarPA in different image grades of RD-ILD. Kruskal-Wallis rank sum test were used in multiple samples; Bonferroni adjustment used for pairwise comparisons. ****p < 0.0001, ***p < 0.001, **p < 0.01, *p < 0.05, NS:p>0.05.

Consistent with findings from previous studies, our findings showed that the serum levels of anti-CarPA in patients with RA, SLE, and pSS were 34.40 ± 32.96 ng/ml (p <0.0001), 21.12 ± 22.23 ng/ml (p = 0.005), and 16.32 ± 13.54 ng/ml (p = 0.005), respectively, which were higher than those of HCs (7.30 ± 5.05 ng/ml). Moreover, the anti-CarPA titers were higher in patients with PM/DM (30.85 ± 17.34 ng/ml, p <0.0001), SSC (23.53 ± 10.70 ng/ml, p = 0.0019), and UCTD (28.35 ± 21.91 ng/ml, p <0.0001) than in HCs. No differences in the mean levels of anti-CarPA was observed between patients with AAV [8.85 (7.05, 32.15) ng/ml, p = 0.10], SpA (14.41 ± 10.70ng/ml, p=0.19), and BD [5.72 (2.73, 9.08) ng/ml, p >1.00] and HCs. As reported previously, the anti-CarPA levels in HCs were comparable (**Figure 2A**).

The clinical varieties of patients with RDs who are anti-CarPA negative and positive were compared. Compared to the RA patients without anti-CarPA, those with anti-CarPA had higher levels of WBC (6.39 ×10^9^/L vs 5.53 ×10^9^/L, p = 0.048), RF (145.50 vs 59.95 KU/ml, p = 0.004), and DAS28 (5.05 vs 4.69, p=0.047) (**Table 2A**). In SLE, patients who were anti-CarPA-positive had higher levels of ESR (55.24 vs 35.68 mm/h, p = 0.006), rRNP (58.65 vs 17.22 RU/ml, p = 0.006), U1RNP (464.60 vs 17.05 CU/ml, p = 0.048), and SLEDAI [6.50 (4.75, 8.00) vs 4.00 (3.00, 7.00), p=0.036] (**Table 2B**). Anti-CarPA-positive patients with PM/DM had a high prevalence of arthralgia (p = 0.034) (**Table 2C**). Among anti-CarPA-positive patients with pSS, PLT levels were not frequently decreased (275.29 ×10^9^/L vs 193.05 ×10^9^/L, p = 0.026) (**Table 2D**). Additionally, the levels of autoantibodies and inflammatory markers in patients with SSC and UCTD did not differ significantly between the anti-CarPA-positive and negative groups.

Table 2Clinical and laboratory characteristics of patients with RDs.

**Table:** 

A. Clinical and laboratory characteristics of patients with RA grouped by anti-CarPA.				
RA	Total	Positive	Negative	P
WBC(10^9^/L(IQR))	6.09(5.12,8.15)	6.39(5.59,9.09)	5.53(4.76,7.91)	0.048*
CRP(median(IQR),mg/ml)	12.70(3.23,32.85)	17.15(4.49,62.05)	7.87(1.55,25.49)	0.086
ESR(mean ± SD,mm/h)	47.99 ± 31.35	52.68 ± 34.52	43.52 ± 27.73	0.20
RF(median(IQR),KU/ml)	85.00(36.45,333.00)	145.50(52.40,420.25)	59.95(3.20,194.50)	0.004*
CCP(median(IQR),U/ml)	195.10(47.48,200.00)	200.00(71.70,200.00)	172.55(27.50,200.00)	0.19
MCV(median(IQR),U/ml)	378.45(58.33,1000.00)	493.55(66.33,1000.00)	227.80(24.58,1000.00)	0.21
ILD(n(%))	12/78(15.38%)	8/38(21.05%)	4/40(10.00%)	0.18
DAS28(mean ± SD)	4.87 ± 1.00	5.05 ± 0.84	4.69 ± 1.12	0.047*

**Table:** 

B. Clinical and laboratory characteristics of patients with SLE grouped by anti-CarPA.				
SLE	Total	Positive	Negative	P
WBC(median(IQR),10^9/L)	4.27(2.91,7.68)	4.06(2.72,8.44)	4.47(2.89,7.28)	0.58
Hb(mean ± SD,g/L)	106.76 ± 22.79	106.12 ± 17.78	107.05 ± 24.93	0.98
PLT(mean ± SD,10^9/L)	196.66 ± 99.02	226.41 ± 106.30	183.21 ± 93.98	0.090
ESR(mean ± SD,mm/h)	41.72 ± 25.82	55.24 ± 28.59	35.68 ± 22.32	0.006*
CRP(median(IQR),mg/ml)	6.07(0.87,22.10)	9.22(2.62,26.6)	3.61(0.66,19.32)	0.094
C3(median(IQR),g/L)	0.72(0.49,0.99)	0.72(0.56,1.04)	0.77(0.48,1.00)	0.94
C4(mean ± SD, g/L)	0.13 ± 0.11	0.13 ± 0.12	0.13 ± 0.10	0.96
dsDNA(median(IQR),IU/ml)	75.19(21.87,310.59)	219.89(56.78,455.12)	47.35(16.83,288.98)	0.11
SM(median(IQR),CU)	19.30(6.00,123.10)	39.30(6.80,428.05)	15.65(5.25,54.90)	0.13
AnuA(median(IQR),RU/ml)	14.77(4.78,59.18)	17.90(2.50,171.61)	12.95(4.46,49.34)	0.24
SSA(median(IQR),RU/ml)	75.14(6.95,175.33)	161.94(12.40,200.0)	67.01(5.22,153.96)	0.052
rRNP(median(IQR),RU/ml)	30.03 ± 56.34	58.65 ± 73.67	17.22 ± 41.73	0.006*
U1RNP(median(IQR),CU)	75.00(3.90,643.80)	464.60(45.10,643.80)	17.05(3.10,643.80)	0.048*
Skin rash(n(%))	18/63(28.57%)	4/18(22.22%)	14/45(31.11%)	0.35
Arthralgia(n(%))	34/63(53.96%)	11/18(61.11%)	23/45(51.11%)	0.33
Raynaud phenomenon(n(%))	16/63(25.39%)	3/18(16.67%)	13/45(28.89%)	0.25
Lupus nephritis (n(%))	14/63(22.22%)	5/18(27.77%)	9/45(20.00%)	0.36
ILD(n(%))	14/63(22.22%)	5/18(27.77%)	9/45(20.00%)	0.36
SLEDAI(median(IQR))	5.00(3.00,8.00)	6.50(4.75,8.00)	4.00(3.00,7.00)	0.036*

**Table:** 

C. Clinical and laboratory characteristics of patients with PM/DM grouped by anti-CarPA.				
PM/DM	Total	Positive	Negative	P
CK(median(IQR),U/L)	179.00(48.00,865.00)	178.00(48.25,1163.50)	190.00(30.50,649.00)	0.63
CRP(median(IQR),mg/ml)	4.50(0.89,13.68)	4.41(0.78,12.15)	13.77(0.65,32.26)	0.56
ESR(median(IQR),mm/h)	29.00(8.00,56.00)	53.00(7.50,74.50)	26.00(8.75,47.75)	0.32
FER(median(IQR),ng/ml)	475.97(41.00,942.00)	482.52(137.25,1381.24)	246.66(94.41,765.95)	0.22
Skin rash(n(%))	30/53(56.60%)	18/34(52.94%)	12/19(63.16%)	0.39
Arthralgia(n(%))	20/53(37.74%)	17/34(50.00%)	3/19(15.79%)	0.034*
Skeletal muscle weakness(n(%))	32/53(60.38%)	22/34(64.71%)	10/19(52.63%)	0.56
ILD(n(%))	37/53(69.81%)	27/34(79.41%)	10/19(52.63%)	0.11

**Table:** 

D. Clinical and laboratory characteristics of patients with pSS grouped by anti-CarPA.				
pSS	Total	Positive	Negative	P
PLT(mean ± SD,10^9/L)	213.61 ± 95.97	275.29 ± 94.15	193.05 ± 89.44	0.026*
CRP(median(IQR),mg/ml)	1.26(0.48,1.93)	1.49(0.69,2.99)	1.04(0.41,1.72)	0.14
ESR(median(IQR),mm/h)	26.5(12.0,64.5)	36.00(12.00,98.00)	25.00(11.00,49.50)	0.10
SSA(median(IQR),RU/ml)	134.43(2.42,183.93)	168.40(48.89,184.34)	123.07(1.20,183.87)	0.59
SSB(median(IQR),RU/ml)	3.33(0.00,109.37)	3.99(2.50,19.83)	2.41(0.00,150.98)	0.86
Raynaud phenomenon(n(%))	8/32(25.00%)	3/10(30.00%)	5/22(22.73%)	0.68
Arthralgia(n(%))	12/32(37.50%)	6/10(60.00%)	6/22(27.27%)	0.12
ILD(n(%))	7/32(21.88%)	4/10(40.00%)	3/22(13.64%)	0.17

SD, standard deviation; IQR, interquartile range; WBC, white blood cell; Hb, hemoglobin; PLT, platelet; ESR, erythrocyte sedimentation rate; CRP, C reaction protein; RF, rheumatoid factor; CCP, anti-cyclic citrullinated peptide antibody; MCV, anti-mutated citrullinated vimentin antibody; ILD, Interstitial lung disease; DAS28, Disease Activity Score 28; C3, complement 3; C4, complement 4; dsDNA, anti-dsDNA antibody; SM , SM antibody; AnuA, anti-nucleosome antibody; SSA, antibodies reactive against the ribonucleoprotein antigens Ro/Sjögren’s syndrome A; SSB, antibodies reactive against the ribonucleoprotein antigens La/Sjögren’s syndrome B antigen; rRNP, anti-ribosomal antibody; U1RNP, anti-U1 small ribonucleoprotein antibody; SLEDAI, SLE Disease Activity Index; CK, creatine kinase.

Parametric data results were expressed as mean ± SD values. Nonparametric data results were expressed as median IQR values. Some values presented as n or n%; n represents the number of people with this clinical manifestation. T-test, Mann-Whitney U tests and Chi-square test are used. *p<0.05; Student’s-test was used in comparison between parametric data. Mann-Whitney test was used in comparison between parametric data and nonparametric data or nonparametric data.

### Association between anti-CarPA and RD-ILD

Eighty-seven patients with ILD were identified in this cohort. Compared to those without ILD, patients with ILD had higher serum levels of anti-CarPA (33.41 vs 22.51 ng/ml, p = 0.0002). Anti-CarPA was found to be higher in ILD groups of RA, PM/DM, and pSS (RA-ILD vs RA-non-ILD: 49.65 vs. 28.17 ng/ml, p = 0.014; PM/DM-ILD vs PM/DM-non-ILD 34.37 vs 23.81 ng/ml, p = 0.045; pSS-ILD vs. pSS-non-ILD, 26.15 vs 13.57 ng/ml, p = 0.027) than in the non-ILD group. In patients with SLE, SSC, AAV, and UCTD, however, anti-CarPA titers were not significantly different between the ILD and non-ILD groups (**Figure 2B**). Additionally, we examined the relationship between anti-CarPA and the radiographic features, including the image classification and image grading, in patients with RD-ILD. The image classification of RD-ILD seemed not to be significantly associated with anti-CarPA levels (UIP: 32.52 ng/ml, NSIP: 35.81 ng/ml, unclassifiable: 31.10 ng/ml, p = 0.62) (**Figure 2C**). Additionally, among the subgroups of ILD, differences in serum levels of anti-CarPA were observed (grade I: 21.90 ng/ml, grade II: 38.16 ng/ml, and grade III: 41.61 ng/ml, p = 0.0027). In pairwise comparisons, differences were observed between grade I and grade II (p = 0.045) and grade I and grade III (p = 0.003) (**Figure 2D**).

### Association between anti-CarPA and RA-ILD

**Figure 3:**
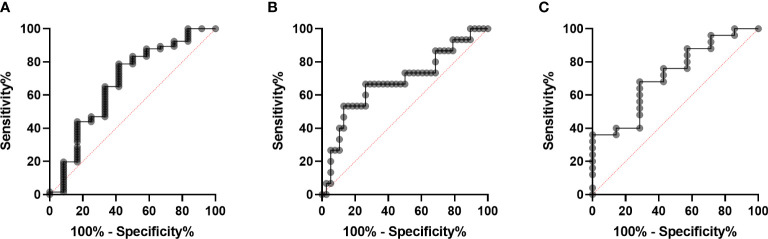
Diagnostic performance of serum anti-CarPA. **(A)** Receiver operating characteristic (ROC) curve analysis of serum anti-CarPA for diagnosis of RA-ILD[area under curve (AUC) 0.67, 95%CI 0.49-0.86)]; **(B)** ROC curve analysis of serum anti-CarPA for diagnosis of PM/DM-ILD (AUC 0.68, 95%CI 0.51-0.85); **(C)** ROC curve analysis of serum anti-CarPA for diagnosis of pSS-ILD (AUC 0.72, 95%CI 0.51-0.93).

The differences in clinical and laboratory features between RA-ILD and RA-non-ILD patients are shown in our results. Patients with RA-ILD had older age (67.67 vs 52.65 years, p=0.001) and a higher level of CRP than RA-non-ILD patients (53.63 vs 8.43 mg/ml, p = 0.004). In terms of other laboratory markers, such as disease duration, ESR, CCP, RF, and MCV, no significant difference was observed (**Table 3A**). To further exclude the effects of clinical confounders, single factor regression was performed. A factor could be added to a multi-factor regression with only p <0.1 in single factor regression. In this study, age, gender, smoking, and CRP were all considered covariates in RA. According to the logistic regression model adjusted for covariates, anti-CarPA was independently associated with RA-ILD (OR: 1.02, 95% CI: 1.00–1.04, p = 0.033) (**Table 3B**). Additionally, we examined the association between disease course and RA-ILD, ESR, RF, CCP, and MCV and found no significant association. The results of our study demonstrated no significant differences in CCP, MCV, or RF, depending on ILD status. The results were not altered after adjusting for clinical confounding factors. To determine whether serum anti-CarPA could act as a diagnostic biomarker of ILD among patients with RA, receiver operating characteristic (ROC) curve analysis was performed, resulting in an AUC of 0.860 for RA-ILD. The serum anti-CarPA >32.59 ng/ml was used as a cut-off value to diagnose ILD in patients with RA, with a sensitivity and specificity of 78.79% and 58.33%, respectively (**Figure 3A**).

Table 3Analysis of the risk factors for patients with RA-ILD.

**Table:** 

A. Clinical and laboratory characteristics of RA patients grouped by ILD.							
RA		Total		RA-ILD	RA-non-ILD		P
Gender(n,M/F)		16/62		5/7	11/55		0.063
Age(mean ± SD,years)		54.96 ± 14.47		67.67 ± 9.21	52.65 ± 14.09		0.001*
Disease duration(median(IQR),month)		9.50(3.00,60.00)		24.00(6.00,105.00)	8.50(3.00,51.00)		0.23
Smoker(n(%))		7/78(8.97%)		3/12(25.00%)	4/66(6.06%)		0.069
WBC(median(IQR),10^9/L)		6.09(5.12,8.45)		6.70(5.67,10.74)	5.93(5.08,8.05)		0.13
CRP(median(IQR)mg/ml)		12.69(3.23,32.85)		53.63(19.57,83.20)	8.43(2.60,27.94)		0.004*
ESR(mean ± SD,mm/h)		47.98 ± 31.35		57.25 ± 34.66	46.30 ± 30.70		0.27
CCP(median(IQR),U/ml)		195.10(47.48,200.00)		143.50(52.95,200.00)	200.00(44.68,200.00)		0.59
RF(median(IQR),KU/ml)		85.00(36.45,333.00)		52.45(18.43,485.50)	87.35(41.88,333.00)		0.45
MCV(median(IQR),U/ml)		378.45(58.33,1000.00)		232.95(59.43,1000.00)	408.00(54.73,1000.00)		0.81
Anti-CarPA(mean ± SD,ng/ml)		31.47 ± 28.04		49.65 ± 44.68	28.17 ± 22.84		0.014*

**Table:** 

B. Binary logistic regression analysis of patients with RA-ILD.							
Single factor regression	OR	95%CI	P	Multi-factor regression	OR	95%CI	P
Anti-CarPA	1.02	1.00-1.04	0.028*	Anti-CarPA	1.02	1.00-1.04	0.033*
Age	1.11	1.04-1.23	0.003*	Age	1.14	1.04-1.25	0.010*
Gender	3.57	0.96-13.34	0.058	Gender	1.29	0.21-7.88	0.78
Disease duration	–	–	0.41	–	–	–	–
Smoke	0.41	0.70-2.41	0.32	Smoke	0.24	0.016-3.69	0.36
CRP	1.02	1.00-1.03	0.020*	CRP	1.01	0.99-1.02	0.24
ESR	–	–	0.27	–	–	–	–
RF	–	–	0.95	–	–	–	–
CCP	–	–	0.79	–	–	–	–
MCV	–	–	0.95	–	–	–	–

SD, standard deviation; IQR, interquartile range; WBC, white blood cell; ESR, erythrocyte sedimentation rate; CRP, C reaction protein; RF, rheumatoid factor; CCP, anti-cyclic citrullinated peptide antibody; MCV, anti-mutated citrullinated vimentin antibody; ILD, Interstitial lung disease; RA-ILD, interstitial lung disease associated with rheumatoid arthritis; 95%CI, 95% confidence interval.

Parametric data results were expressed as mean ± SD values. Nonparametric data results were expressed as median IQR values. Some values presented as n or n%; n represents the number of people with this clinical manifestation. T-test, Mann-Whitney U tests, Chi-square test and Logistic regression analysis are used. *p<0.05.

### Association between anti-CarpA and PM/DM-ILD

The differences in clinical and laboratory features between PM/DM-ILD and PM/DM-non-ILD patients are summarized in **Table 4**. Patients with PM/DM-ILD had older age (55.61 vs 49.00 years, p = 0.022), longer disease duration (8.00 vs 2.00 months, p = 0.005), and higher levels of CRP (7.32 vs 1.21 mg/ml, p = 0.012) than PM/DM-non-ILD patients (**Table 4A**). Age and smoking status, which were selected by a single factor regression and a previous study, were controlled as confounders in the PM/DM group. An analysis of multi-factor logistic regression demonstrated that anti-CarPA is independently associated with PM/DM-ILD (OR: 1.05, 95% CI: 1.00–1.10, p = 0.040) (**Table 4B**). However, ESR, CRP, and autoantibodies, including anti-threonyl-transfer ribonucleic acid synthetase anti-body (anti-PL-7), anti-glycyl-transfer ribonucleic acid synthetase antibodies (anti-EJ), anti-histidyl-transfer ribonucleic acid synthetase antibody (anti-JO-1), anti-melanoma differentiation-associated gene 5 antibodies (anti-MDA-5), and antibodies of Ro-52 (anti-Ro-52), showed no significant difference in association with PM/DM-ILD. Following this, Spearman’s correlation analysis was applied to determine if different MSAs were associated with anti-CarPA. According to our study, the levels of anti-CarPA were negatively correlated with anti-transcription intermediary factor 1 complex antibodies (anti-TIF1) (R = –0.28, p = 0.044), while positively correlated with anti-EJ (R = 0.30, p = 0.031), and anti-MDA-5 (R = 0.35, p = 0.011). No association between other MSAs and anti-CarPA were observed. Additionally, ROC of anti-CarPA was generated for patients with PM/DM-ILD, with serum anti-CarPA >23.46 ng/ml (AUC: 0.68, p = 0.038, sensitivity: 66.67%, specificity: 73.68%) being the optimal cutoff (**Figure 3B**).

Table 4Analysis of the risk factors for patients with PM/DM-ILD.

**Table:** 

A. Clinical and laboratory characteristics of PM/DM patients grouped by ILD.							
PM/DM		Total	PM/DM-ILD		PM/DM-non-ILD		P
Gender (n,M/F)		19/34	3/12		16/22		0.13
Age(mean ± SD,years)		54.24 ± 10.34	55.61 ± 9.51		49.00 ± 12.65		0.022*
Disease duration(median(IQR),month)		6.00(2.00,12.00)	8.00(3.75,13.5)		2.00(1.00,3.00)		0.005*
Smoker(n(%))		14/53(26.42%)	10/38(26.32%)		4/15(26.67%)		0.62
CK(median(IQR),U/L)		190.00(48.00,956.00)	179.00(34.00,1019.00)		217.50(55.50,1155.75)		0.073
CRP(median(IQR),mg/ml)		4.50(0.89,13.68)	7.32(1.60,15.60)		1.21(0.00,6.64)		0.012*
ESR(median(IQR),mm/h)		29.00(8.00,56.00)	44.00(7.00,67.00)		17.00(8.25,24.5)		0.11
FER(median(IQR)ng/ml)		475.97(120.17,1005.58)	578.06(182.45,1024.94)		153.93(96.06,583.74)		0.36
Anti-CarPA(mean ± SD,ng/ml)		31.43 ± 2.41	34.37 ± 2.82		23.81 ± 4.24		0.045*

**Table:** 

B. Binary logistic regression analysis of patients with PM/DM-ILD.							
Single factor regression	OR	95%CI	P	Multi-factor regression	OR	95%CI	P
Anti-CarPA	1.00	1.00-1.09	0.052	Anti-CarPA	1.05	1.00-1.10	0.040*
Age	1.06	1.00-1.12	0.033*	Age	1.07	1.01-1.14	0.029*
ender	2.91	0.70-12.03	0.14	–	–	–	–
Disease course	1.00	1.00-1.00	0.33	–	–	–	–
Smoke	1.02	0.26-3.40	0.98	Smoke	0.87	0.20-3.90	0.85
Gender	1.04	0.98-1.10	0.20	–	–	–	–
ESR	1.01	0.99-1.04	0.27	–	–	–	–
Anti-PL-7	1.84	0.30-11.22	0.51	–	–	–	–
Anti-EJ	–	–	1.00	–	–	–	–
Anti-JO-1	–	–	1.00	–	–	–	–
Anti-MDA-5	1.86	0.86-4.01	0.12	–	–	–	–
Anti-Ro-52	1.45	0.86-2.43	0.16	–	–	–	–

SD, standard deviation; IQR, interquartile range; CK, creatine kinase; ESR, erythrocyte sedimentation rate; CRP, C reaction protein; FER, ferritin; anti-PL-7, anti-threonyl-transfer ribonucleic acid synthetase anti-body; anti-EJ, anti-glycyl-transfer ribonucleic acid synthetase antibody; anti-JO-1, anti-histidyl-transfer ribonucleic acid synthetase antibody; anti-MDA-5, anti-melanoma differentiation-associated gene 5 antibody; anti-Ro-52,anti-Ro52 antibody; ILD, Interstitial lung disease; PM/DM-ILD, interstitial lung disease associated with polymyositis/dermatomyositis; 95%CI, 95% confidence interval.

Parametric data results were expressed as mean ± SD values. Nonparametric data results were expressed as median IQR values. Some values presented as n or n%; n represents the number of people with this clinical manifestation. T-test, Mann-Whitney U tests, Chi-square test and Logistic regression analysis are used. *p<0.05.

### Association between anti-CarPA and pSS-ILD

The clinical and laboratory characteristics of patients with pSS grouped by ILD show a higher level of PLT in the pSS-ILD patients than in the pSS-non-ILD patients (**Table 5A**). The binary logistic regression analysis showed a relationship between pSS-ILD and anti-CarPA. Single factor logistic regression suggested that anti-CarPA was independently associated with pSS-ILD (OR = 1.07, 95% CI: 1.00–1.10, p = 0.048). From single factor logistic regression, no risk factor could be adjusted (**Table 5B**). ROC curve of anti-CarPA in pSS-ILD showed that the best cut-off for anti-CarPA in pSS-ILD was a level >37.08 ng/ml (AUC: 0.773, p = 0.040, sensitivity: 33.33%, specificity: 96.00%) (**Figure 3C**).

Table 5Analysis of the risk factors for patients with pSS.

**Table:** 

A. Clinical and laboratory characteristics of pSS patients grouped by ILD.				
pSS	Total	pSS-ILD	pSS-non-ILD	P
Gender (n,M/F)	31/1	1/6	0/25	0.22
Age(mean ± SD,years)	53.11 ± 13.06	55.50 ± 6.35	52.45 ± 14.40	0.052
Disease duration(median(IQR),month)	30.00(6.00,84.00)	24.00(6.00,36.00)	36.00(6.00,84.00)	0.71
PLT(mean ± SD,10^9/L)	213.61 ± 95.97	310.50 ± 83.07	187.18 ± 82.38	0.002*
CRP(median(IQR),mg/ml)	1.26(0.48,1.93)	1.36(0.38,3.37)	1.17(0.50,1.80)	0.39
ESR(median(IQR),mm/h)	26.50(12.00,64.50)	24.50(9.25,101.0)	26.50(12.00,55.50)	0.88
SSA(median(IQR),RU/ml)	134.43(2.43,183.93)	105.02(0.00,180.07)	134.43(2.47,184.52)	0.60
SSB((median(IQR),RU/ml)	3.33(0.00,109.37)	2.59(0.00,115.82)	5.08(0.00,117.28)	0.67
Anti-CarPA(mean ± SD,ng/ml)	16.30 ± 2.42	26.15 ± 6.61	13.57 ± 2.20	0.027*

**Table:** 

B. Binary logistic regression analysis of patients with pSS-ILD.				
Single factor regression		OR	95%CI	P
Anti-CarPA		1.07	1.00-1.14	0.048*
Age		1.01	0.95-1.08	0.69
Gender		–	–	1.00
Disease course		1.00	1.00-1.00	0.64
CRP		1.28	0.71-2.30	0.41
ESR		1.00	0.99-1.03	0.52
SSA		1.00	0.99-1.01	0.64
SSB		1.00	0.99-1.01	0.71

SD, standard deviation; IQR, interquartile range; PLT, platelet; ESR, erythrocyte sedimentation rate; CRP, C reaction protein; SSA, antibodies reactive against the ribonucleoprotein antigens Ro/Sjögren’s syndrome A; SSB, antibodies reactive against the ribonucleoprotein antigens La/Sjögren’s syndrome B antigen; ILD, Interstitial lung disease; pSS-ILD, interstitial lung disease associated with primary Sjögren’s syndrome; 95%CI, 95% confidence interval.

Parametric data results were expressed as mean ± SD values. Nonparametric data results were expressed as median IQR values. Some values presented as n or n%; n represents the number of people with this clinical manifestation. T-test, Mann-Whitney U tests and Chi-square test are used.*p<0.05.

## Discussion

Anti-CarPA was shown to be widely distributed among patients with RDs in this study. Past studies have shown that anti-CarPA persists in parts of RDs: Verheul et al. (1) enrolled more than 5,000 patients with RA, and the positive rate of anti-CarPA was 34–53%. Nakabo et al. (14) included 241 patients with SLE in Japan, and the positive rate of anti-CarPA was 54.4%. Two studies from Europe showed that the positive rates of anti-CarPA in patients with pSS were 26.9% and 30%, respectively (15, 18). Riccardi et al. (17) included 448 French patients with SSC, and the positive rate of anti-CarPA was 14%. These studies showed that anti-CarPA were not the specific antibody for RA, which inspired us it may exist in various RDs. Besides, the clinical value of anti-CarPA in different RDs remained unclear, which should be identified whether it related to occurrence and development of some manifestations in RDs. Currently, the research object of previous studies were mainly among Caucasian, but the research on the distribution of anti-CarPA among the Chinese Han population remains insufficient. Therefore, we conducted this study among the Chinese Han population to explore the distribution and clinical significance of anti-CarpA. Our study confirmed the presence of anti-CarpA in several RDs, including RA, SLE, pSS, SSC, PM/DM, UCTD, SpA, and BD. In our study, anti-CarPA was detected in 48.70% of patients with RA, 28.57% of patients with SLE, 31.25% of patients with pSS, 62.26% of patients with PM/DM and 55.00% of patients with UCTD, further confirming and expanding these findings. As described above, preliminary results indicated that anti-CarPA antibodies are broadly distributed and have a low specificity in RDs. According to our observations, the serum titer of anti-CarpA was obviously higher in RA than in other RDs. Furthermore, anti-CarPA was proven to be detectable almost 14 years before RA appeared (36). Anti-CarPA seemed to play a significant role in the diagnosis and prediction of RA, especially among patients with RA-ILD.

In terms of clinical and laboratory variables, anti-CarPA-positive and negative patients were quite different in several aspects. Patients with anti-CarPA positive RA had younger onset ages and longer disease durations, which were consistent with previous observations (3, 22, 37). In spite of these findings, we observed that RA patients with positive anti-CarPA had higher levels of RF and DAS28, which potentially resulted in more severe joint damage (38, 39), and could indicate poor prognosis. A significant increase in ESR and SLEDAI were observed in SLE individuals with positive anti-CarPA in our study. Li et al. (19) observed that anti-CarPA was associated with high disease activity in SLE patients. It was consistent with our results, and suggested that anti-CarPA was associated with disease activity in patients with SLE. Massaro and Ceccarelli (40, 41) confirmed that anti-CarPA was associated with joint damage in patients with SLE. However, our results showed no significant difference between anti-CarPA and arthralgia. It could be accounted for that joint ultrasonography was not a regular examination for SLE patients in our department, so that patients with joint damage in early stage, especially patients with mild symptom, were prone to miss in this study. In the PM/DM group, we observed an association between anti-CarPA and arthralgia (p = 0.034). The most common finding in joint damage of idiopathic inflammatory myopathies (IIM) is active synovitis, which is similar to RA (42). Highly abundant neutrophils in synovium have been observed to be capable of forming neutrophil extracellular traps (NETs) that externalize carbamylated autoantigens to the extracellular space, resulting in an increase in the production of anti-CarPA (43, 44), which in turn causes joint inflammation. Anti-CarPA may play a role in arthritis associated with PM/DM in a manner similar to RA. However, further validation is required. The correlation between anti-CarPA and RA-ILD has been proposed in recent years (23, 45, 46). Our study identified for the first time that serum anti-CarPA was upregulated in various patients with RD-ILD, not only RA but also PM/DM and pSS. This upregulation was associated with the severity of pulmonary fibrosis but not the image classifications of HR-CT. Anti-CarPA may act as a biomarker for predicting RA-ILD, suggesting more serious lung involvement.

An association seemed to exist between anti-CarPA and ILD in patients with PM/DM in our study. We found that PM/DM-ILD patients had higher levels of CRP than patients with PM/DM-non-ILD. Accordingly, CRP may be involved in the development and progression of PM/DM-ILD. Gono (47) developed a prediction model termed MCK (MDA5, CRP, and KL-6) to identify patients with PM/DM-ILD at low, moderate, or high risk of mortality, using CRP as a risk factor. In our study, we identified an association between anti-CarPA and PM/DM-ILD and proved a relationship between anti-CarPA and severer RD-ILD. Therefore, we speculate that anti-CarPA participates in PM/DM-ILD development and progression, whereas little evidence exists on how anti-CarPA contributes to PM/DM-ILD. In spite of this finding, anti-CarPA is positively correlated with anti-MDA5 and anti-EJ, while negatively correlated with anti-TIF-γ in patients with PM/DM. Several studies found that anti-MDA5 and anti-EJ were associated with more severe ILD in patients with PM/DM, whereas anti-TIF-γ was related to less lung involvement (48). Previous studies confirmed that NETs could participate in the development of ILD in IIM (49–51). Seto (51) identified that anti-MDA5 promotes the formation of NETs, in turn to induce epithelial cell injury and inflammatory cytokine release. In RA, NETs seem to play a role in the production of anti-CarPA, whereas in ILD, the neutrophils that contribute to the production of NETs increase near the lesion site (43). Anti-CarPA is believed to play an important role in the progression of PM/DM-ILD.

Bergum et al. (18) have confirmed that anti-CarPA was associated with the severity of pSS. Our study interestingly found that PLT was lower in anti-CarPA-negative subgroups of pSS. Wu et al. (52) have observed that patients with pSS without thrombocytopenia were more likely to have ILD. Our observations suggest rather that an association exists between them. Additionally, we showed that levels of anti-CarPA were correlated with pSS-ILD and it could predict pSS-ILD. Furthermore, our study found no significant difference in serum anti-CarPA levels between patients with SSC with and without ILD. In a recent study, Riccardi et al. (17) showed an association between anti-CarPA with “fibrotic subset” in patients with SSC, including patients with diffuse cutaneous subset and/or interstitial lung disease. A study with a large sample size is needed to confirm these findings. The SLE-ILD and UCTD-ILD groups had no association with anti-CarPA, indicating that different pathogenetic mechanisms may be involved.

In this study, we extend the study of anti-CarPA to examine its widespread distribution in RDs among Chinese Han nationals. Moreover, it is the first study to examine the relationship between anti-CarPA and RD-ILD, especially non-RA-ILD. According to our results, anti-CarPA plays an important role in RD-ILD and could be used to identify patients with RDs who are at high risk of developing ILD. This study had two main limitations. First, the relative small number of patients enrolled was the main study limitation. Therefore, further studies on a larger population are mandatory to clarify the prognostic value of anti-CarPA in patients with RDs, especially for patients with RD-ILD. Second, the triage system in our country made it likely that our cohort would enroll patients with more severe diseases. However, a series of analyses have indicated that anti-CarPA is associated with several RDs and linked with ILD in patients with RA, PM/DM, and pSS.

## Conclusion

For the first time, we demonstrated the presence of anti-CarPA in a Chinese cohort of patients with RDs, such as RA, SLE, PM/DM, pSS, and UCTD. Based on the results of our study, anti-CarPA may assist in the identification of ILD in patients with RA, PM/DM, and pSS. Further replicative investigations may confirm the pathologic role of anti-CarPA in patients with RDs. Serum anti-CarPA could be detected in patients with RA, PM/DM, pSS, SSC, and UCTD among the Chinese. And it may also assist in identifying ILD in patients with RA, PM/DM, and pSS, which emphasized attention to the lung involvement in anti-CarPA-positive patients.

## Data availability statement

The raw data supporting the conclusions of this article will be made available by the authors, without undue reservation.

## Ethics statement

The studies involving human participants were reviewed and approved by Shandong Provincial Hospital validated the study protocol (SZRJJ: NO.2021-438). Written informed consent to participate in this study was provided by the participants’ legal guardian/next of kin.

## Author contributions

RD and YS participated in the study design, literature review, and performed statistical analysis and presentation of the results and participated in the drafting and review of the manuscript. RD, WX, WZX, and ML collected serum samples. ZM, QY, and LZ participated in the study design, the drafting and review of the manuscript. All authors contributed to the article and approved the submitted version.
